# Full-mouth rehabilitation with implant-supported overdentures in a heavy smoker with multiple sclerosis: A 3-year follow-up case report

**DOI:** 10.34172/japid.025.3973

**Published:** 2025-10-15

**Authors:** Rola Muhammed Shadid

**Affiliations:** ^1^Department of Prosthodontics, Faculty of Dentistry, Arab American University, Jenin, Palestine; ^2^Private Practice, Tulkarm, Palestine

**Keywords:** Attachment, Bar overdenture, Case report, Implant, Multiple sclerosis, Smoking

## Abstract

This case report describes the rehabilitation of a 70-year-old Arab male patient with relapsing-remitting multiple sclerosis (MS), controlled type 2 diabetes, and heavy smoking. The patient was treated with a maxillary four-implant bar-supported overdenture and a mandibular two-implant tissue-supported overdenture chosen for their stability, minimal invasiveness, affordability, and ease of hygiene. At three-year follow-up, peri-implant bone levels remained stable, and function and quality of life improved. However, moderate inflammation and plaque accumulation reflected fair oral hygiene and persistent smoking. This case emphasizes the need for strict maintenance at three-month intervals and elimination of risk factors, particularly smoking and poor glycemic control. Implant therapy in MS patients should be undertaken cautiously with comprehensive risk assessment and interdisciplinary planning. Although this single case showed favorable outcomes, the findings should be interpreted with caution, given the persistent high-risk factors and limited generalizability.

## Introduction

 Multiple sclerosis (MS) is a chronic inflammatory neurological disorder characterized by immune-mediated damage to myelin and oligodendrocytes in the central nervous system, leading to sclerotic plaques.^1,2^ It develops in genetically susceptible individuals exposed to environmental triggers such as infections, vitamin D deficiency, smoking, and Epstein-Barr virus.^3,4^ MS usually manifests in early adulthood, affects women three times more than men, and its global prevalence is rising, with about 2.8 million cases worldwide.^5-7^

 Clinical features depend on plaque location and include muscle weakness, ataxia, paralysis, sensory loss, visual disturbances, and urinary or cognitive dysfunction.^8-11^ Orofacial manifestations are common in these patients and include trigeminal neuralgia, dysarthria, oral pain, xerostomia, and increased risk of caries, periodontitis, and temporomandibular disorders.^5,10,12-14^ Medications may also add oral complications such as candidiasis, xerostomia, gingival hyperplasia, and even malignancies.^15,16^

 Diagnosis relies on clinical signs, MRI, and cerebrospinal fluid analysis.^10^ Management includes supportive care and disease-modifying therapies such as steroids, interferons, immunosuppressors, and biologics.^8,17,18^

 Oral health complications directly affect dental treatment planning in patients with MS. For edentulous patients, dental implants can improve prosthesis retention, oral function, and quality of life. Implant-retained overdentures represent a preferred treatment option, offering functional rehabilitation with reduced surgical morbidity, easier hygiene maintenance, and lower cost.

 The goal of this case report is to contribute to the limited literature on dental implant rehabilitation in patients with MS. Only one case report of implant therapy in MS has been identified, without long-term follow-up.^8^ To the best of the author’s knowledge, the present case is among the very few documented prosthodontic rehabilitation cases with implant-supported overdentures in an MS patient with a three-year follow-up.

## Case Report

###  Ethics

 Before clinical examinations and treatment, the patient provided informed consent acknowledging the risks of implant surgery in the context of his MS diagnosis, heavy smoking, and diabetes. Alternatives, including conventional mucosa-borne prostheses, were explained with their advantages and disadvantages. Additional written consent was obtained for publication of this case report and accompanying images.

###  Clinical Examination

 A 70-year-old Arab man presented to the author’s private clinic in Palestine with the request to chew and smile without pain. He had been diagnosed with relapsing-remitting type MS at the age of 35. He reported blurred vision and wore eyeglasses, was limping, and complained of tingling sensations in the skin and anxiety. The patient was also a well-controlled type 2 diabetic, with an HbA1c of 7 measured within three months of implant surgery, and reported smoking 20 cigarettes per day for 20 years (20 pack-years).

 He was prescribed Copaxone (glatiramer acetate) (40 mg/mL) three times per week, administered subcutaneously, Gabapentin (800 mg daily), and Januet XR (100 mg/1000 mg) (sitagliptin and metformin) daily. He had not been hospitalized or undergone any surgery in the last three years.

 Extraoral examination focusing on the temporomandibular joint (TMJ), facial symmetry, and possible trigger points of trigeminal neuralgia was within normal limits.

**Figure 1:**
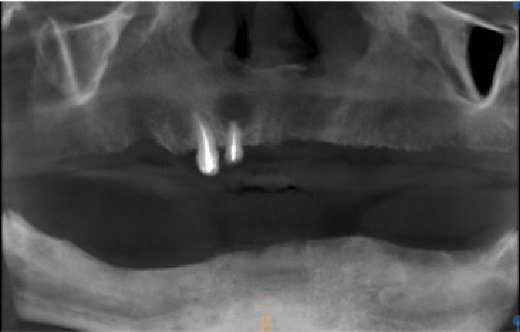
Preoperative panoramic view of CBCT

**Figure 2:**
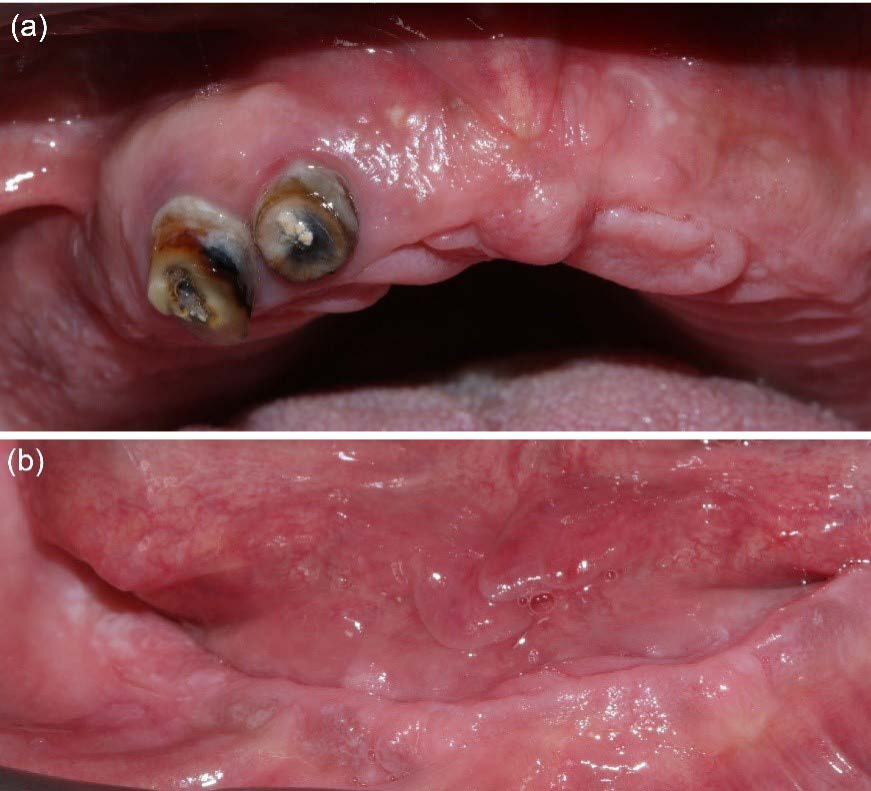
Preoperative clinical views of the maxillary (a) and mandibular (b) arches

 Intraoral examination revealed complete edentulism in the mandible and partial edentulism in the maxilla with carious retained roots of the right lateral incisor and canine. Localized gingival inflammation with plaque and calculus accumulation was evident (Figures 1, 2a, 2b). He reported brushing his remaining teeth irregularly—no more than twice weekly—and rarely using mouthwash, and that he was unable to accept the transitional maxillary removable partial denture and the mandibular complete denture.

###  Treatment Planning

 Following clinical and radiographic assessment, different options were proposed. However, the patient expressed a desire for an affordable treatment that would minimize surgical invasiveness and allow easier cleaning. His supervising physician also recommended placing the fewest possible implants. Consequently, the treatment plan consisted of a maxillary four-implant bar-supported overdenture and a mandibular two-implant tissue-supported overdenture. The final attachment system included a CAD/CAM-milled titanium splinting bar with four locator attachments and a cobalt–chromium reinforcement structure in the maxilla, and two individual equator attachments in the mandible.

###  Implant Surgery

 After extracting the two remaining maxillary roots, implant placement was scheduled eight weeks later. Prophylaxis included Augmentin, 2 g 1 hour preoperatively, which continued for 7 days, as well as 0.12% chlorhexidine rinses twice daily, starting 1 day before surgery and continuing for 2 weeks postoperatively. The patient was advised to cease smoking 1 week before surgery and for at least 8 weeks afterward; he admitted to reducing smoking to 2‒3 cigarettes per day during the critical healing period.

 In the maxilla, surgery was carried out under local anesthesia. Bilateral midcrestal incisions were made, preserving the incisive papilla and extending posteriorly to the first molar regions. After reflection of full-thickness mucoperiosteal flaps, limited osteoplasty was performed to level the ridge, as there was already approximately 14 mm of restorative space from the fitting surface of the previous denture to the incisal plane, which was deemed sufficient for the planned implant overdenture.^19^ After osteotomies were completed, four MIS C1 conical connection implants (3.75 × 11.5 mm, 3.75 × 13 mm, 3.3 × 11.5 mm, and 3.75 × 11.5 mm) were inserted with an insertion torque of 30–40 Ncm. Cover screws were placed, and the flaps were sutured with 4-0 vicryl.

**Figure 3:**
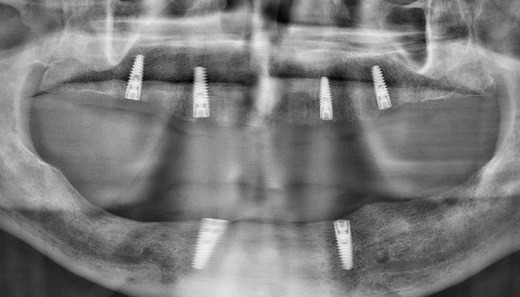
The panoramic radiograph after implant placement

 In the mandible, a lingually positioned crestal incision with a vertical midline releasing incision was performed. After raising a full-thickness flap and minor osteoplasty, two sites were prepared (measuring 7 mm) on each side of the midline. The osteoplasty was performed on the left side to level the ridge, since there was already about 12 mm of restorative space measured from the fitting surface of the complete denture to the incisal plane, which was deemed sufficient for the planned implant overdenture.^19^ Two MIS C1 implants (3.75 × 13 mm) were placed in the lateral incisor/canine regions with 50-Ncm torque, and cover screws were installed. Sutures were placed, hemostasis was achieved, and a panoramic radiograph confirmed the implant positions (Figure 3). Healing was uneventful, and sutures were removed after 14 days.

 At 16 weeks, second-stage surgery was carried out. In the maxilla, apically positioned partial-thickness flaps were used, and in the mandible, a small midcrestal incision was made. Implant stability quotients ranged from 67 to 73. Straight multi-unit abutments were connected to three maxillary implants, while the left canine implant received a 17° angled multi-unit abutment. Healing abutments were attached to mandibular implants.

###  Prosthodontic Procedures

 Six weeks after second-stage surgery, soft tissue healing was satisfactory, and fabrication of definitive prostheses commenced. Custom trays were fabricated with openings over the implant sites, border molding was performed with heavy body polyvinylsiloxane, and open tray impressions were taken with regular body material after splinting impression copings with dental floss and light-cured composite resin. Impressions were poured using pink silicone and type IV dental stone.

 Record bases with occlusion rims were used to establish esthetics, occlusal vertical dimension, and centric relation. Facebow transfer was performed, and casts were mounted on a semi-adjustable articulator. Restorative space measurements confirmed 14 mm for the maxilla and 12 mm for the mandible. Ivoclar acrylic resin teeth were arranged in bilateral balanced occlusion and tried in the mouth to evaluate esthetics, phonetics, and centric relation.

**Figure 4:**
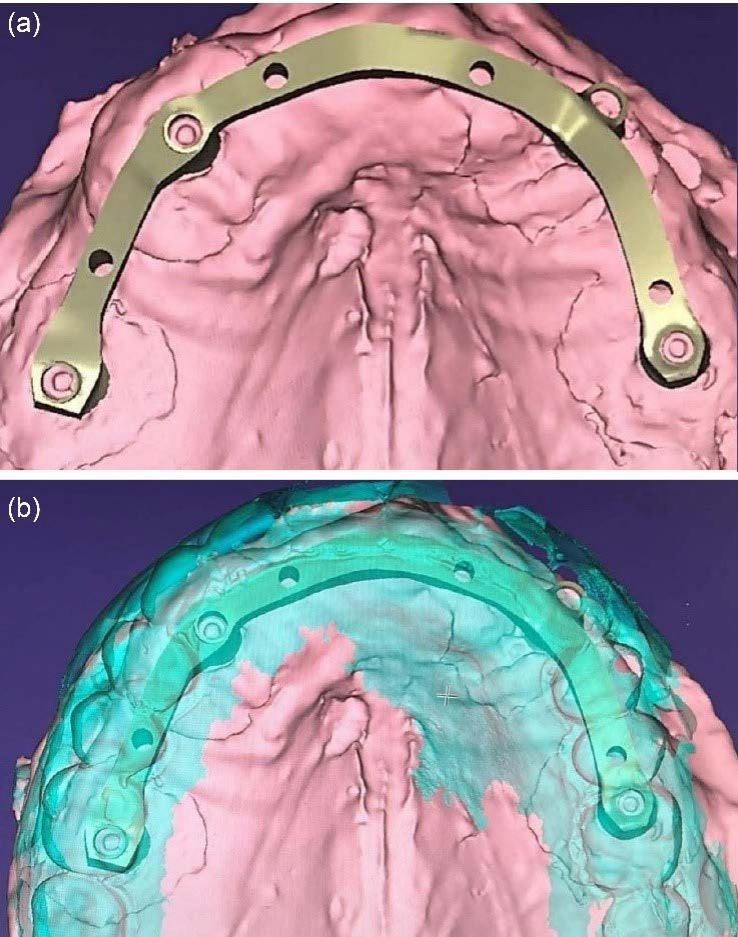
Tentative maxillary CAD/CAM splinting bar design (a) and positioning within denture teeth setup (b), showing four locator attachments

**Figure 5:**
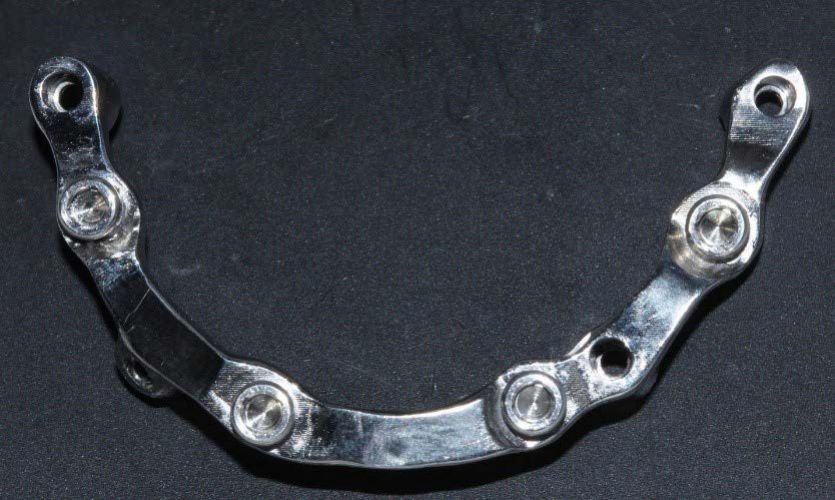
Laboratory occlusal view of the maxillary CAD/CAM bar with four incorporated locator attachments

**Figure 6:**
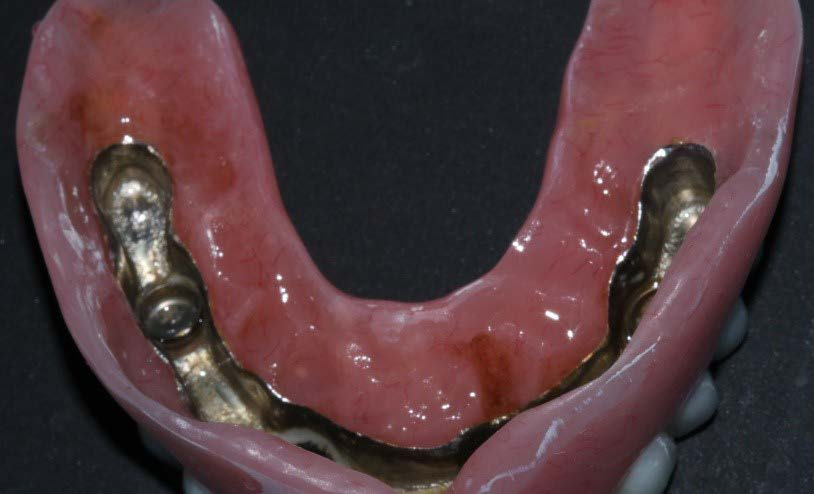
Intaglio surface of horseshoe maxillary overdenture with cobalt–chromium minibase

 For the maxilla, the wax denture and cast were scanned, and a CAD/CAM titanium bar was designed to fit within the contours of the denture, incorporating locator attachments (Figures 4a, 4b). The locator attachments were screwed into the tapped threads of the milled bar to 20-Ncm torque (Figure 5), and the bar was verified intraorally for passive fit using the Sheffield one-screw test and radiographs. A cobalt–chromium reinforcement minibase with integrated housings was fabricated, and the dentures were processed with heat-cured acrylic resin. Since the anteroposterior spread was 20 mm and implant lengths were sufficient, a palateless design was selected (Figure 6).^20^

**Figure 7:**
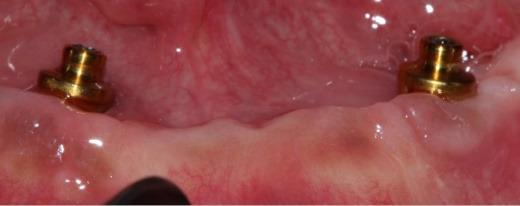
Clinical view of two mandibular implants with equator attachments in place

**Figure 8:**
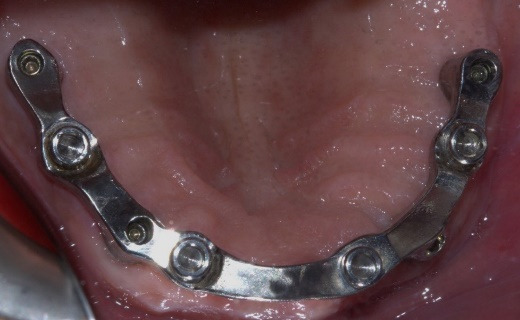
Occlusal view of maxillary CAD/CAM splinting bar with four locator attachments screwed in place, providing retention, stability, and support to the overdenture

**Figure 9:**
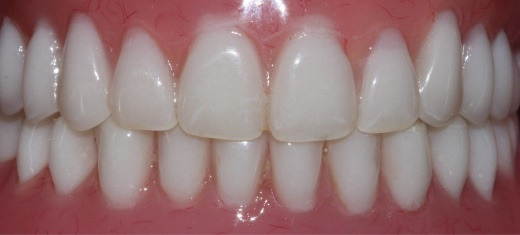
Clinical anterior view with overdentures in place

**Figure 10:**
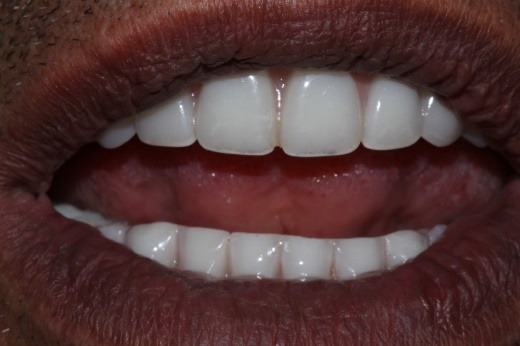
Extraoral close-up of the patient smiling with maxillary and mandibular overdentures in place

**Figure 11:**
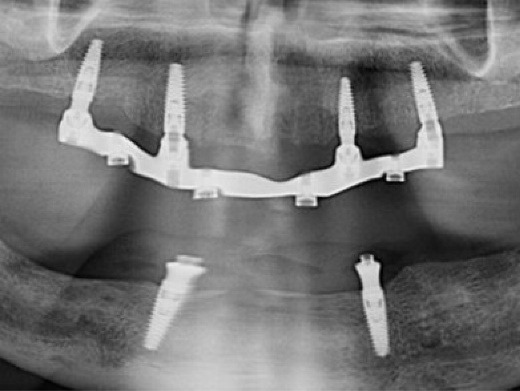
Panoramic radiograph immediately after prosthetic placement

 At delivery, mandibular healing abutments were removed and replaced with OT-Equators torqued to 30 Ncm, while maxillary bar screws were torqued to 25 Ncm in the maxilla (Figures 7, 8). Chairside pick-up of housings seated on mandibular equators was completed with autopolymerizing resin, and occlusion was adjusted to bilateral balanced contacts (Figures 9, 10.). Black nylon inserts, the least retentive type, were kept in the housings of both the maxillary and mandibular overdentures at the patient’s request, and replacement with more retentive inserts was deferred. Final panoramic radiographs were obtained (Figure 11).

###  Follow-up and Maintenance

 The patient received instructions on oral hygiene, including the use of manual and electric toothbrushes and oral irrigators, and was advised to remove overdentures at night. He was informed about the need to replace nylon inserts approximately every six months and to replace overdentures every 5–7 years.

**Figure 12:**
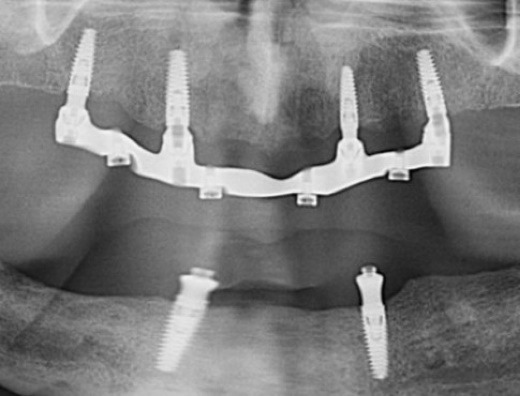
Panoramic radiograph 3 years after prosthetic treatment

 Follow-up appointments were scheduled at 24 hours, 1 week, and 3 months post-insertion, and every 3 months thereafter. Up to three years after delivery, radiographic evaluation showed stable peri-implant bone levels (Figure 12). Clinically, however, the peri-implant soft tissues exhibited moderate inflammation, bleeding on probing, and plaque accumulation, consistent with fair to poor hygiene. Nylon inserts were replaced three times during this period. The patient reported improved chewing, smiling, and social comfort, expressing satisfaction with the treatment and its positive impact on daily life. He reduced smoking to a minimal level during the first 12 months of follow-up but later resumed heavy smoking. At each recall, oral hygiene instructions, smoking cessation, and glycemic control maintenance were reinforced. He was strongly advised to follow a smoking cessation protocol and reminded of the adverse effects of smoking on the long-term maintenance of implants.

Table 1 summarizes the chronological sequence of diagnostic, surgical, and prosthetic procedures.

**Table 1:** Case report timeline, according to CARE guidelines

**Time point**	**Clinical event**	**Notes**
Age 35	Diagnosis of relapsing-remitting multiple sclerosis	Treated with Copaxone
Age 50	Diagnosis of type 2 diabetes	Well controlled (HbA1c ≈7); managed with Januet XR
~20 years before implant therapy	Onset of heavy smoking habit	20 cigarettes/day (~20 pack-years)
Baseline (pre-surgery)	Preoperative assessment	Heavy smoking and controlled diabetes documented; medications: Copaxone, Gabapentin, Januet XR
Surgery (Month 0)	Implant placement	4 implants maxilla, 2 implants mandible. Patient advised to cease smoking; reduced intake to 2–3 cigarettes/day during healing period
Month 6	Prosthetic rehabilitation	Maxillary bar overdenture and mandibular implant-retained overdenture delivered
Month 12	Follow-up	Smoking resumed at the previous level; glycemic control remained good; peri-implant bone levels stable
Month 36	Final follow-up	Peri-implant bone levels stable; patient satisfaction high; glycemic control remained good. However, plaque accumulation, bleeding on probing, and moderate inflammation reflected fair to poor oral hygiene.

## Discussion

 This case report illustrates the surgical and prosthetic management of a patient with relapsing-remitting MS, complicated by controlled type 2 diabetes and ongoing heavy smoking. The treatment consisted of a maxillary four-implant bar-supported overdenture and a mandibular two-implant tissue-supported overdenture. At the three-year follow-up, clinical and radiographic outcomes demonstrated stable peri-implant bone levels, satisfactory prosthesis function, and the patient reported improved quality of life.

 The literature search identified only one reported case of dental implant placement in an MS patient, with no follow-up provided.^8^ This case involved a 40-year-old female with relapsing-remitting MS who received three implants to replace the mandibular right first and second molars and the left first molar in Saudi Arabia. However, no data on implant survival, success, or follow-up were reported.^8^This highlights the scarcity of evidence regarding long-term implant outcomes in MS patients and underscores the value of the present report.

 Patients with MS frequently present with trigeminal neuralgia, oral and perioral paresthesia, dysarthria, xerostomia, periodontal disease, and caries,^5,10,12-14^ as well as visual impairment,^11^ muscle weakness, and sensory disturbances.^9^ These conditions, along with the progressive course of MS, complicate oral rehabilitation and necessitate careful risk assessment with interdisciplinary collaboration between prosthodontists, surgeons, and neurologists. Patients with advanced MS and severe spasms often cannot tolerate lengthy dental procedures, require assistance in the dental chair, and may struggle with oral hygiene, making them unsuitable for extensive implant therapy.^21^ In the present case, the disease was stable, and muscle spasms decreased, with a minimum number of implants.

 The selection of overdentures was based on the patient’s request for a less costly option, the physician’s recommendation for minimal invasiveness, and the recognition that overdentures are easier to clean than fixed prostheses in patients with reduced dexterity.^8^ The patient’s orofacial muscular control and manual skills were sufficient to manage removable prostheses. For these reasons, a mandibular two-implant tissue-supported overdenture and a maxillary four-implant bar overdenture were provided.^22^ However, the patient was advised to consider future conversion to a fully implant-supported mandibular overdenture, since two-implant overdentures are associated with the greatest posterior bone loss compared with other designs.^23^

 To reduce the risk of complications, prophylactic antibiotics were prescribed, and submerged healing with extended healing time was selected despite high primary stability, given the patient’s use of immunosuppressive medication,^16,24^ controlled diabetes,^25^ and smoking habit.^26^ Surgery was staged in two separate morning appointments to minimize fatigue, which is common in MS patients.^24^

 Diabetes did not compromise implant outcomes in this case, as HbA1c was maintained at 7, and close glycemic control was maintained. Literature confirms that implant therapy is safe and predictable in well-controlled diabetes, with complication rates comparable to those of healthy individuals.^25^ However, these patients remain at a higher long-term risk of peri-implant inflammation.^25^

 Smoking presents an even greater risk; smokers have been reported to show up to a 140% increase in implant failure and delayed osseointegration compared to nonsmokers.^26^ In light of this evidence, the short-term satisfactory outcome observed in this patient, despite his resumption of heavy smoking, should be interpreted with caution. It may represent an exception rather than the rule and underscores the importance of strict smoking cessation protocols to improve the predictability of implant therapy in similar medically compromised patients.

 At 3 years, peri-implant bone levels remained stable, and patient satisfaction was high. Nevertheless, plaque accumulation, bleeding on probing, and moderate inflammation were observed, reflecting fair to poor oral hygiene. The patient was reminded that meticulous oral hygiene is essential for implant survival and may also help prevent MS exacerbation. He was further counseled to maintain glycemic control and stop smoking.

 The association between MS and periodontal health deserves emphasis. A recent systematic review showed that periodontitis is significantly more prevalent in MS patients than in healthy controls.^5^ Moreover, patients with chronic periodontitis are nearly twice as likely to develop MS,^5^ suggesting that neurodegenerative disease may progress more rapidly in the presence of chronic oral infection.^27^

 Given that only one other implant case in an MS patient has been reported without follow-up,⁸ the present outcome offers additional documentation but should still be interpreted with caution. Stability over three years in a medically compromised MS patient who resumed heavy smoking is infrequently documented in the literature and should not be generalized. Regular three-month recalls, reinforcement of hygiene, and strict control of modifiable risks such as smoking and diabetes remain essential for improving predictability and long-term outcomes in similar patients.

## Conclusion

 This report describes the 3-year rehabilitation of a patient with MS, heavy smoking, and controlled diabetes using maxillary bar-supported and mandibular implant-retained overdentures. Despite persistent systemic and behavioral risk factors, the treatment in this single case resulted in stable peri-implant bone levels and improved oral function. Regular three-month maintenance visits were recommended, given fair oral hygiene and continued smoking. Implant therapy in MS patients who smoke should be undertaken cautiously with thorough interdisciplinary assessment, and additional studies are required before practical recommendations can be made for this population.

## Competing Interests

 The authors declare that they have no competing interests regarding the authorship and/or publications of this paper.

## Consent for Publication

 Written informed consent was obtained from the patient for the publication of this case report and any accompanying images.

## Data Availability

 All data supporting the findings of this case report are included within the article.

## Ethical Approval

 Not Applicable.
